# Vomiting, electrolyte disturbance, and medications; the perfect storm for acquired long QT syndrome and cardiac arrest: a case report

**DOI:** 10.1186/s13256-021-03204-7

**Published:** 2022-01-11

**Authors:** K. D. Tiver, D. Dharmaprani, J. X. Quah, A. Lahiri, K. E. Waddell-Smith, A. N. Ganesan

**Affiliations:** 1 Department of Cardiology, Level 6, Flinders Medical Centre, Flinders Drive, Bedford Park, SA 5042 Australia; 2 College of Medicine and Public Health, Flinders University, Bedford Park, Australia

**Keywords:** Acquired long QT syndrome, Torsades de pointes, Ondansetron, Fluoxetine, Metoclopramide, Hypokalemia, Hypomagnesemia, Renewal theory, Case report

## Abstract

**Background:**

Acquired long QT syndrome is an important and preventable cause of cardiac arrest. Certain medications and electrolyte disturbance are common contributors, and often coexist. In this case, we report five contributors to cardiac arrest.

**Case presentation:**

This case is of a 51-year-old Caucasian female patient who presented with vomiting associated with hypokalemia and hypomagnesemia. She subsequently received ondansetron and metoclopramide, on the background of chronic treatment with fluoxetine. She then suffered an in-hospital monitored cardiac arrest, with features of long QT and torsades de pointes retrospectively noted on her prearrest electrocardiogram. She was diagnosed with acquired long QT syndrome, and her QT interval later normalized after removal of offending causes.

**Conclusions:**

This case highlights the importance of proper consideration prior to prescribing QT prolonging medications, especially in patients who have other risk factors for prolonged QT, such as electrolyte disturbances and pretreatment with QT prolonging medications.

## Background

Long QT syndrome (LQTS) is complex; a genetically, physiologically, and environmentally multifactorial disorder of cardiac ion channels, which leads to abnormal cardiac repolarization. It is, therefore, a cause of ventricular arrhythmias (VAs), in particular torsades de pointes (TdP) and sudden cardiac death (SCD) [1]. Although much of the interest of cardiologists and researchers is focused on genetic LQTS [2], acquired LQTS has also been recognized as being as “risky” as congenital LQTS [3], and is vastly more common in clinical practice [4]. The prevalence of acquired LQTS is estimated to be 25–30% in hospitalized populations [5], and most commonly occurs in noncardiac units [6]; however, the true incidence of acquired LQTS causing death is likely to be underreported.

Acquired LQTS is commonly caused by medications [7], electrolyte abnormalities [8], structural heart disease, bradyarrhythmias, starvation, hypothermia, toxins, endocrine disease, liver disease, human immunodeficiency virus infection, and inflammation [9]. It is estimated that 5–7% of reports of VAs or SCD are due to drug-induced LQTS and TdP [10]. The “multi-hit theory” suggests that more than one risk factor is required to cause clinical or electrocardiographic manifestations of acquired LQTS [4, 11]. One case series of 11 patients with acquired LQTS revealed all patients had electrolyte abnormalities (including severe hypokalemia), and an average of 2.8 QT prolonging medications administered [12]. In a study of patients admitted to hospital with QT prolongation, 35% of them received QT prolonging medications, commonly leading to further prolongation of the QT interval [5], indicating a genuinely under-appreciated issue. Many cases of TdP can be prevented by careful prescribing [13].

## Case presentation

**Fig. 1:**
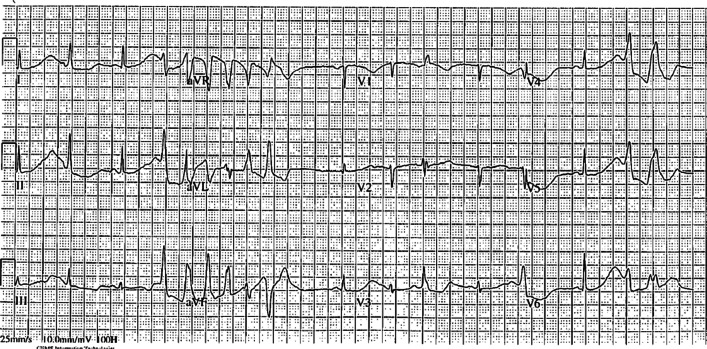
Electrocardiogram just prior to cardiac arrest, demonstrating very long QT interval, T wave alternans, and short runs of torsades de pointes

**Fig. 2:**
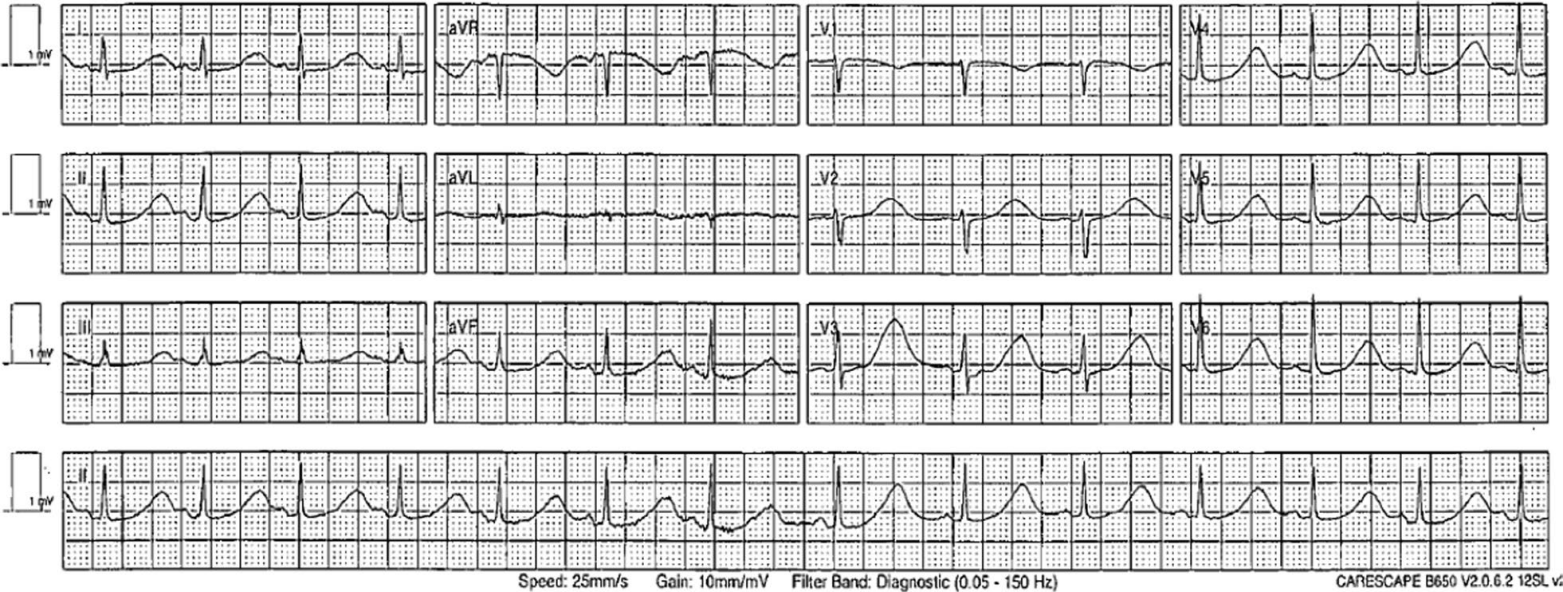
Electrocardiogram following resuscitation, demonstrating significantly prolonged QT interval

**Fig. 3:**
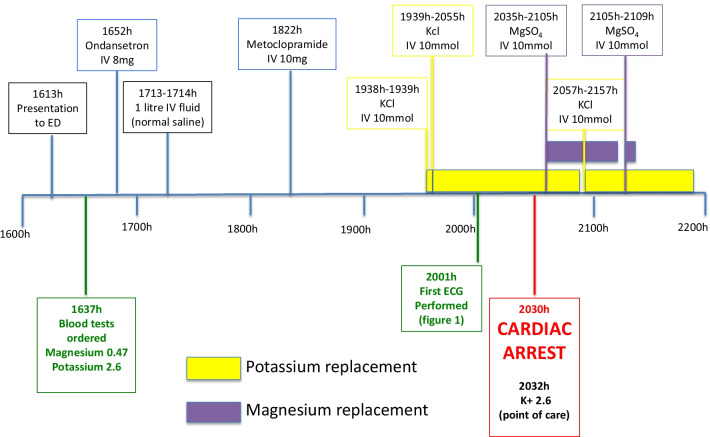
Fishbone diagram of presentation, investigations, medications, and infusions administered. *ED* emergency department, *KCl* potassium chloride, *IV* intravenous, *MgSO*_*4*_ magnesium sulfate

A 51-year-old Caucasian woman presented to the emergency department with several hours of recurrent vomiting, associated with abdominal cramping but no diarrhea. There was no history of unwell contacts or culprit dietary intake, nor was there a history of fevers or any symptoms to suggest another focus of infection. There was no hematemesis or melena. She had no history of illicit drug use. Her past medical history included depression, treated with fluoxetine 20 mg, orally, daily. She had no cardiac history, and no significant family history of sudden cardiac death. She was managed with antiemetics, including ondansetron and metoclopramide, and intravenous fluid rehydration with normal saline. Clinical examination demonstrated dehydration, without any apparent cause of the vomiting detectable on clinical examination. Investigations revealed hypokalemia (2.5 mmol/L) and hypomagnesemia (0.47 mmol/L), and replacement was commenced. Her initial electrocardiogram (ECG), showed sinus rhythm, with corrected QT interval (QTc) of 680 msecond and had nonsustained runs of TdP and T wave alternans (Fig. 1). She then had a witnessed cardiac arrest, with the presenting rhythm of polymorphic ventricular tachycardia. She received 30 seconds of chest compressions and was successfully resuscitated without requirement for defibrillation, and recovered to a normal neurologic baseline. Her postresuscitation ECG (Fig. 2) showed a prolonged QT interval. Her postresuscitation venous blood gas showed a pH of 7.304, bicarbonate of 20.3 mmol/L, sodium of 142 mmol/L, potassium of 2.6 mmol/L, and glucose of 7.5 mmol/L. The sequence of events is represented in Fig. 3. She underwent further cardiac structural assessment, which was normal. Investigations into alternative causes of the vomiting, including septic screening, liver and kidney function testing, and exclusion of pancreatitis did not reveal a specific cause, and her vomiting resolved spontaneously. She was presumed to have had a viral illness. Her QT normalized after several days of observation, following withdrawal of offending medications and replacement of electrolytes, and she was discharged home with a diagnosis of acquired long QT syndrome. At follow up clinic review at 2 months and at 12 months, she remained asymptomatic with a normal QT interval, and will have ongoing follow-up.

## Discussion

This case report highlights the “perfect storm” of electrolyte disturbance (potassium, magnesium) and medication effects (fluoxetine, ondansetron, and metoclopramide), leading to acquired long QT syndrome and cardiac arrest.

Ondansetron is known to prolong the QT interval in a dose-dependent manner [14], it is recommended to be used with caution in patients who may develop prolongation of QTc, such as patients with electrolyte abnormalities [15]. Of note, it is very likely for this scenario to occur, as vomiting is not only the indication for administering ondansetron, but is also a risk factor for electrolyte abnormalities and, therefore, QT prolongation.

Fluoxetine is also known to cause prolonged QT interval. The product information suggests it should be used with caution with other clinical conditions that predispose to arrhythmias, such as hypokalemia or hypomagnesemia [16]. Based on a recent comparative review, fluoxetine was reported to have one of the lower risk profiles of its class for causing QT prolongation, along with sertraline and fluvoxamine [17]. Prescription of selective serotonin reuptake inhibitors (SSRIs) is so common in the community that even a rare side effect will be observed. In 2018–2019, 17.1% of the Australian population received a mental health-related prescription, 70.9% of these were antidepressants, and SSRIs are first line antidepressants [18]. These rates are increasing with time, indicating a growing percentage of our population with a baseline risk factor for QT prolongation, even prior to acute illness.

Although metoclopramide on its own is not listed as a QT prolonging medication, and no warnings about QT prolongation exist on its product information, there is evidence that it can contribute to QT prolongation. There are reports of important pharmacokinetic and pharmacodynamic drug–drug interactions that can contribute to QTc prolongation, especially with ondansetron [19]. In addition, metoclopramide has been mechanistically shown to change QT dynamics [20], as well as showing a signal in epidemiological research [21]. It has been added to crediblemeds.org as a “TdP conditional drug” [22], meaning that in combination with other medications, it may prolong the QT interval and increase the risk of TdP.

Despite the good clinical outcome in this case, mortality of cardiac arrest survivors remains high, even when the underlying cause was transient or correctable [23]. Some authors have suggested implantable defibrillators may beneficial, even in this situation of an acquired proarrhythmic state [24]. The patient presented in this case was assessed to have acquired long QT syndrome, rather than congenital long QT syndrome based on the normalization of the QT interval following removal of the five precipitating factors. Following removal of the precipitants, the patient’s Schwartz score for diagnosis of congenital LQTS was 0, indicating low risk of the congenital condition. Given the tendency demonstrated towards prolonged QT with precipitating conditions, however, she will have ongoing clinical electrophysiology review to monitor her symptoms and QT interval.

The patient in this case feels she did not understand what was happening to her, or why it was happening. She does fear another cardiac arrest, which is impacting on her life.

In this case, we make the observation that TdP is self-limited after a number of seconds in the first ECG, with no patient symptoms, and subsequently only a few minutes later, TdP is prolonged, leading to the devastating consequence of cardiac arrest. We do not currently understand what causes TdP to self-terminate, or to continue [1]. Perhaps this could be understood by studying TdP from a wavefront dynamic perspective, given the observations that QRS morphology often abruptly switches from positive to negative during the warm-up phenomenon [11]. A characteristic feature of TdP is that the vast majority of polymorphic ventricular arrhythmia episodes are self-terminating, as observed in this case. The mechanisms underlying this repetitive pattern of self-termination, and what distinguishes TdP from more sustained forms of ventricular fibrillation, are currently unclear. Our group has recently extensively been studying arrhythmia self-termination in atrial fibrillation and ventricular fibrillation. In self-terminating epochs, it appears that the rate of reentrant rotor formation and destruction is much slower compared with more sustained arrhythmia epochs. We have developed a novel statistical framework based on renewal theory to characterize these differences [25–27]. It would be of significant interest to extend these approaches to the clinical scenario of TdP, which would allow us to gain insight into the optimal prophylactic and treatment strategies for this form of arrhythmia.

## Conclusion

This case serves as an important reminder that vomiting leads to both electrolyte disturbances and the prescription of antiemetics, which here led to a quadruple hit (hypokalemia, hypomagnesemia, ondansetron, metoclopramide) causing TdP and cardiac arrest. SSRIs are so commonly prescribed, that 1 in 6 patients may have this preexisting risk factor for TdP, as this patient did, contributing to the multiple hits required for clinical manifestations of acquired LQTS. This case occurred in a monitored hospital emergency room, and the patient had a favorable outcome. However, there is a genuine risk of fatal outcomes from a similar situation for a patient in the ambulatory setting, where patients can present with vomiting and are often given antiemetics without performing biochemistry or clinical observation.

## Take home message

This case illustrates the clinical teaching point that care must be taken to avoid multiple “hits” that contribute to QT prolongation. The commonly available antiemetics have all been associated with QT prolongation to some extent, so if antiemetic therapy is needed in the future for this patient, care must be taken to check electrolytes and monitor the QT interval during treatment. In such a patient, SSRI treatment in the future should be considered on a risk versus benefit basis. If indicated, fluoxetine, along with sertraline and fluvoxamine are least QT prolonging SSRIs. These may be given, but careful attention must be paid to monitoring the QT interval and avoiding other risk factors for QT prolongation.

## Data Availability

Not applicable.

## Abbreviations

LQTS: Long QT syndrome
VA: Ventricular arrhythmias
TdP: Torsades de pointes
SCD: Sudden cardiac death
ECG: Electrocardiogram
QTc: Corrected QT interval
SSRI: Selective serotonin reuptake inhibitor
ED: Emergency department
KCl: Potassium chloride
MgSO_4_: Magnesium sulfate
